# A Robot-Mediated Activity Using the Nao Robot to Promote COVID-19 Precautionary Measures among Older Adults in Geriatric Facilities

**DOI:** 10.3390/ijerph19095222

**Published:** 2022-04-25

**Authors:** Lauriane Blavette, Anne-Sophie Rigaud, Salvatore Maria Anzalone, Clément Kergueris, Baptiste Isabet, Sébastien Dacunha, Maribel Pino

**Affiliations:** 1 Faculté de Médecine, Université de Paris, Maladie d’Alzheimer, 75006 Paris, France; laurianebla@gmail.com (L.B.); anne-sophie.rigaud@aphp.fr (A.-S.R.); baptiste.isabet@brocalivinglab.org (B.I.); sebastien.dacunha@aphp.fr (S.D.); 2 Services de Gériatrie 1 & 2, AP-HP, Hôpital Broca, 75013 Paris, France; 3 Laboratoire CHArt, Université Paris 8, 93526 Saint-Denis, France; anzalone.s@gmail.com; 4 Broca Living Lab, CEN STIMCO, 75013 Paris, France; clement.kergueris@etu.utc.fr

**Keywords:** older adults, social robot, COVID-19, precautionary measures, health promotion, engagement

## Abstract

Precautionary measures (e.g., social distancing, mask wearing, washing hands regularly) to limit the transmission of the Coronavirus 19 (COVID-19) have been put in place worldwide. However, a limited understanding of precautionary measures and low compliance with them has been observed in older adults with neurocognitive disorders, persons with intellectual disability, or mental illness. The objective of this study is to create and evaluate a robot-mediated activity to deliver information on COVID-19 precautionary measures in an accessible and engaging way using the humanoid robot Nao. An interactive scenario explaining and demonstrating COVID-19 precautionary measures is created using the verbal and non-verbal behaviours of the robot. The scenario (≈5 min) is presented to 124 users of a geriatric hospital, including the following: older patients (n = 45), accompanying persons (n = 39), and health professionals (n = 40). The data regarding perceived usefulness, acceptability, and accessibility are collected using a questionnaire. A video analysis of the participants’ behaviour during the interaction with the robot is performed to examine the quality of engagement in the activity. The results show a good acceptance, satisfaction, and perceived usefulness of the robot-mediated activity. These findings suggest that robot-mediated interventions using humanoid robots can be an effective tool for the delivery of health promotion information.

## 1. Introduction

Persons of all age groups are at risk of contracting Coronavirus Disease 19 (COVID-19) infection. However, older adults (65+ years old) have a significantly higher risk of developing severe forms of the disease due to physiological changes that come with ageing and other potential underlying health issues [1,2]. The presence of major neurocognitive disorders (NCDs), also known as dementia, a frequent comorbidity in this age group, is, for instance, associated with an increased risk of COVID-19 related hospitalisation and mortality [3]. Although vaccination against COVID-19 in older adults has decreased the severity of COVID-19 infections, as evidenced by a reduction in hospitalizations in this population [4], precautionary measures (e.g., mask wearing, regular hand hygiene, keeping a safe distance, sneezing, or coughing in the elbow, etc.) are still important to limit the risk of contagion and thus slow down the epidemic.

Public health organisations around the world have implemented and made mandatory the application of such precautionary measures [5]. Since the beginning of the pandemic, information on precautionary measures has been widely shared by different means (e.g., television, newspapers, the internet, posters, etc.). Nevertheless, for some specific populations, the capacity to access information about COVID-19 precautionary measures and to understand and/or apply these measures is limited. This is often the case for older adults with cognitive impairment, persons with mental illness, cognitive disability, or neurodevelopmental disorders [6,7,8]. In most of these cases, cognitive dysfunction and/or neuropsychiatric symptoms make it difficult to find and effectively use health promotion information as well as to adapt to continuously changing preventive health measures [9].

Some studies have shown that older adults generally respect COVID-19 precautionary measures [10,11]. In contrast, in older adults with major NCDs, compliance with precautionary measures has been shown to be low due to deficits in memory, reasoning, and language, among others, which affect their ability to understand and retain COVID-19 guidelines for disease prevention [6,8,12]. A study conducted in 2020 in a Medical Center for Dementia in Japan showed that most patients with Alzheimer’s disease (61.8%) did not recognise the COVID-19 pandemic and did not wear face masks properly by themselves (74.5%), most likely because of cognitive impairment [13]. Cognitive deficits affect the ability of older adults with NCDs to understand a message, especially if it is discursive in nature [14], or if it uses abstract verbal language [15]. It is therefore essential to find appropriate communication strategies so that older adults with NCDs accept and understand COVID-19 prevention and health monitoring recommendations.

General guidelines for communicating effectively with older adults with NCDs include using simple sentences, speaking clearly and slowly, giving the person enough time to understand and respond, offering simple choices, and using non-verbal communication (e.g., making eye contact, using gestures, facial expressions, body language, etc.), or visual prompts (e.g., cue cards, pictures, videos) to convey meaning [16,17,18].

Many initiatives around the world have been taken to make COVID-19 related information accessible to persons with intellectual or developmental disabilities, such as older adults with NCDs. For instance, in North America, the Centers for Disease Control and Prevention (CDC) has provided a list of accessible resources including guidelines and tools to help people with disabilities protect their health, for instance, as follows: easy-to-read materials, social stories (i.e., using pictures and simple sentences to provide instructions), posters, and video series [19]. In France, the Public Health France organisation provides several accessible resources as well, such as information sheets presenting COVID-19 precautionary measures associated with easy-to-read texts and pictures [20]. Most of these communication materials use the principle of behaviour modelling to encourage the imitation and adoption of preventive measures.

Some researchers from the field of robotics have developed COVID-19 related interventions either to transmit information or to support preventive measures using robots [21]. Robotic systems, which have been used in hospitals, nursing homes, older adults’ homes, and other public places, check, for instance, that masks are worn correctly, social distancing is respected, or if a person has an unusually high temperature, giving a warning accordingly. Studies have shown that communication through robots encourages users’ attention. The robot’s non-verbal behaviour seems to facilitate the persuasion of the information message and to be more effective than the simple use of verbal instructions to convey information.

Social robots are an innovative tool that could be used to provide accessible information on COVID-19 precautionary measures, encourage their imitation, and motivate compliance among the public with intellectual disability or cognitive disorders. The possibility of adapting the robot’s communication style (e.g., simplified dialogues, slow speed of speech, and a positive and playful interactional style) makes them suitable for use by persons who have communication and comprehension difficulties [15]. Humanoid social robots can also be used to model specific behaviours (i.e., using the body gestures of the robot) and positively encourage the user to adopt these behaviours.

In the field of healthcare, various studies have shown the benefits of “robotic mediation” for older adults in geriatric settings. A robot-mediated intervention uses the social robot as an intermediary between the older adult and the therapist or facilitator. A few publications have described the use of humanoid social robots to encourage older adults to participate in adapted physical activity programmes [22,23,24]. These studies have demonstrated that robots can play a motivational role and positively encourage participants to imitate the physical exercises displayed by the robot. Some authors have found that social robots could promote the engagement of older adults with a low level of attention and/or interest in rehabilitation or therapeutic activities [25,26]. Others have described an interest in using a social robot for exercise coaching and information delivery and have indicated that older participants responded better to the gestures of the robot (i.e., embodied cues) than to human instructors or to instructional videos [27,28,29,30]. Globally, all these works have shown a good acceptance of robot-mediated health interventions by older adults and good results in terms of the robot’s ability to promote the imitation of its behaviour by older participants.

The study reported here aimed to develop an interactive informational activity for older adults in geriatric facilities to encourage the learning or recall of COVID-19 precautionary measures, using a humanoid robot as a mediation tool. The robot-mediated activity was intended to be simple, engaging, and accessible. To this end, particular attention was given to aspects facilitating understanding and imitation of precautionary measures (e.g., simple language, playful interaction, modelling gestures). This work was carried out in two stages:

*(1) Phase 1* “*Design and development of the informational scenario*”*:* The objective of this phase was, on the one hand, to define the message on precautionary measures for COVID-19 that would be transmitted during the robot-mediated activity. The accessibility of the message and its relevance to the public of geriatric facilities were central issues at this stage. On the other hand, the verbal and non-verbal behaviours of the robot were defined and programmed to convey the information in an interactive and engaging way using the concept of “embodied cues”. Embodied cues refer to behaviours such as gaze, gestures, proximity, and vocal cues that help encompass meaning. Embodied cues used in human-robot interaction have been shown to contribute to significant social, cognitive, and task outcomes, for instance, improved learning [31].

*(2) Phase 2* “*Pilot assessment of the activity with users and health professionals in a geriatric hospital*”*:* The objectives of this phase were, first, to assess the feasibility, acceptability, and perceived usefulness of the robot-mediated intervention; second, to identify barriers, enablers, as well as recommendations for its implementation. We hypothesised that a robot-mediated intervention would encourage the engagement of participants in a health promotion activity and would support the learning and/or reminding of COVID-19 precautionary measures.

## 2. Methods

### 2.1. Participants

The study was carried out between July and December 2020. The participants in this study were recruited from the following two French geriatric facilities: the Memory Clinic of the Broca Hospital (AP-HP), and the rehabilitation day care hospital of the Vaugirard Hospital (AP-HP). Three groups of participants were recruited: older patients from the geriatric hospitals, accompanying persons, and health professionals from the institutions.

The only inclusion criterion for participation in the study was to be voluntary. No exclusion criteria were defined. The study was approved by the National Comité de Protection des Personnes, CPP Ouest II, Maison de la Recherche Clinique—CHU Angers (Approval Number: 2021/20) and the local ethical committee of Université de Paris (Comité d’Ethique de la Recherche CER—N IRB: 00012020-108) and was compliant with the General Data Protection Regulation (GDPR) (DPO: 20210114153645 AP-HP register).

### 2.2. Material

#### 2.2.1. Nao Robot

This study was carried out with the humanoid robot Nao (V4.0) [32]. This small size (58 cm) and fully programmable robot is equipped with cameras and multiple sensors, which allow the robot a wide range of interactions with its environment. Nao can follow users with its gaze, speak, tell stories, play music, and perform physical actions (e.g., walking, dancing, making gestures with its arms and hands). As a programmable robot, Nao can be used to support a wide range of interactive activities targeting different publics and objectives.

The choice of the robot was based on several arguments. It has 25 degrees of freedom, which allows it to rotate on the different axes (X, Y, and Z) of its neck, arms, and legs [33]. This gives the robot a large possibility of movement. This aspect was relevant to our study, in which one of the objectives was to represent, using the robot, a series of gestures associated with health prevention measures against COVID-19. In addition, Nao’s visual and auditory sensors allow it to follow users with its eyes, favouring interaction [34]. In our project, capturing and maintaining the visual attention of viewers was critical to the delivery of the health prevention message. Nao’s small size allows it to be easily transported from one place to another. In the framework of this study, it was necessary to be able to transport the robot to different locations in order to present the activity to different hospital users. Finally, the Nao robot’s acceptance among OAs has proven to be high by different studies [33].

The modelling of the interactive informational activity was performed using the *Choregraphe* programming tool [35]*. Choregraphe* (Aldebaran Robotics, Paris, France) is a software developed by Softbank Robotics Aldebaran to model pre-recorded or user-created behaviour sequences. This software allows to create all kinds of precise movements for the Nao robot and to provide a wide range of uses [36].

#### 2.2.2. The Informational Robot-Mediated Scenario

An interactive scenario combining robot’s verbal and non-verbal behaviours was developed to present COVID-19 precautionary measures. The content of the prevention message had to be accurate and convincing so that the participants could trust the source of information. For this purpose, preventive measures for COVID-19 were selected based on official public health recommendations [37].

The script was conceived to be simple and accessible to all. A neutral language register was privileged to focus on delivering the facts (not necessarily formal or informal). All the dialogues were tested so that the pronunciation was as clear as possible. The Choregraphe software allows the speed and volume of the Nao’s words to be modulated. In order for the participants to clearly understand the robot, the sound level was set to the maximum (100%) with the possibility to adjust it at any time, following users’ needs. We also slowed down the speech speed of Nao from 100% (normal speech rate) to 75% (slower than normal speech speed).

**Figure 1:**
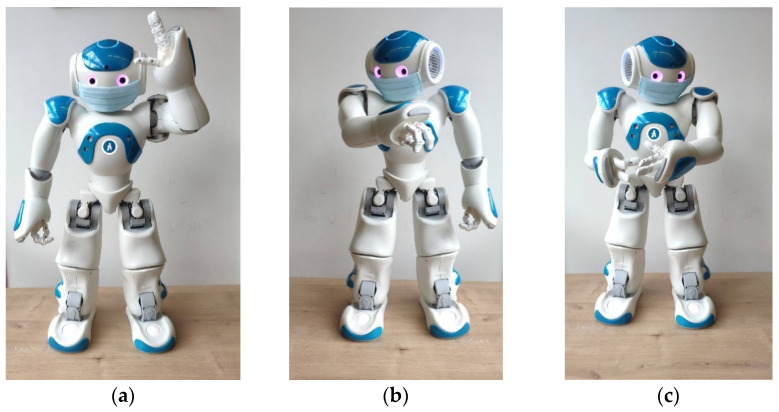
Examples of non-verbal behaviours. (**a**) Nao shows with its hands how to correctly wear a mask; (**b**) Nao sneezes on its elbow; (**c**) Nao shows how to clean hands.

A key point for improving accessibility of the information was to integrate embodied cues into the dialogue, with the aim of giving a stronger meaning to the informational content. In this regard, and to the extent that it was possible, a set of gestures were associated with each verbal explanation of the precautionary measure (e.g., the robot shows with his hands how to correctly put the mask; the robot imitates hand washing; the robot simulates coughing by covering its mouth with its elbow). Moreover, the robot was equipped with a face mask to reinforce the message of prevention. Laughter and sound effects (e.g., sneezing, applause) were also incorporated into the scenario to encourage participants’ attention and motivation. Some examples of non-verbal behaviour of the Nao robot are shown in Figure 1.

Finally, proxemics and the way Nao should move, and approach participants was carefully studied. Using the main categories of inter-individual distances defined by Hall (1963) [38], the demonstration was conceived to start with the robot located at a “social distance” from the participant (i.e., the robot is placed at about 2.50 m/8.2 ft), after introducing itself, the robot should approach the participant to facilitate the visual access to the gestural explanations, up to a “personal distance” (i.e., the robot approaches at a distance of about 1.50 m/4.9 ft). Before moving towards the participant, the robot should warn the person of its action (i.e., “*I’m going to get a little closer to you.*”*)* so as not to take the person by surprise.

**Figure 2:**
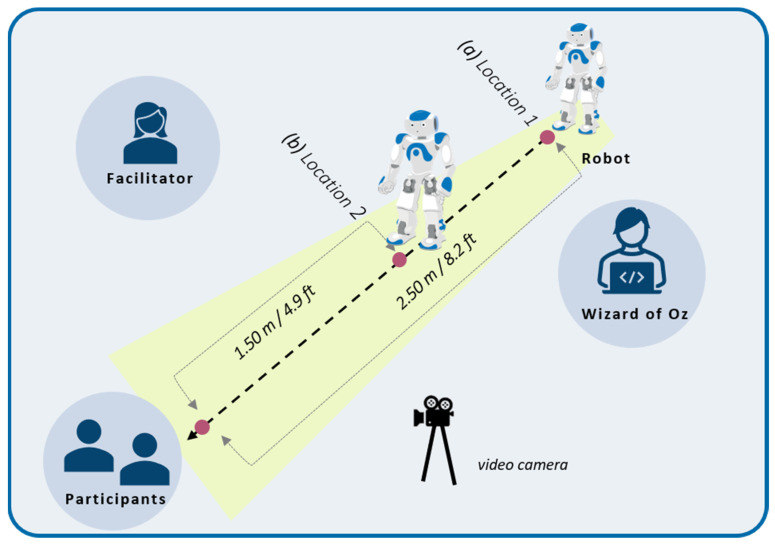
Setting of the experimentation indicating (**a**) the initial location of the robot at the start of the scenario, then its final location (**b**) where it performs the presentation of the precautionary measures.

The robot was positioned on the floor for safety reasons. Even if Nao has a system that allows it to fall safely, a “Fall Manager” [39], it is better to avoid that it falls. Since our activity included multiple movements and gestures that could make the robot fall, we considered that the best option was to position the robot on the floor to avoid further damage in case of a fall. The decision to position the robot on the floor was also examined considering the configuration of the demonstration setting, in which only a small number of people, who were seated, should have visual access to the robot (1–3 maximum), in this regard, the installation of the robot on the floor did not cause any visual access difficulties. Furthermore, it was important that the robot was on the floor level because the scenario included a walking behaviour towards the participant to favour an engagement and the illusion of agentivity among them. The configuration of the experiment space is presented in Figure 2.

To ensure a good quality of content, several versions of the scenario were created and tested iteratively with research and healthcare professionals. The final scenario was composed of the following four sequences:

1. **Introduction.** The robot Nao presents itself as an assistant helping the hospital professionals and says that it will describe five ways to protect oneself from the COVID-19;
2. **Presentation of five precautionary measures.** The robot describes five actions to prevent the transmission of COVID-19. Each explanation is associated to a corresponding gesture. Measures include the following:
  - Correctly wearing a mask;
  - Greeting other people without any physical touch;
  - Maintaining a distance of at least 1.50 m from other people;
  - Sneezing or coughing into the elbow;
  - Cleaning hands frequently (with soap or hydroalcoholic gel);
3. **Summary.** To help participants remember better the precautionary measures, the robot gives a short reminder of the five measures previously presented. Nao asks the participants if they have understood everything or if they want the robot to remind them of certain information. A variable amount of time is then set aside for discussion with the participants;
4. **Conclusion.** The robot Nao concludes the presentation by wishing the participants a pleasant day.

**Figure 3:**
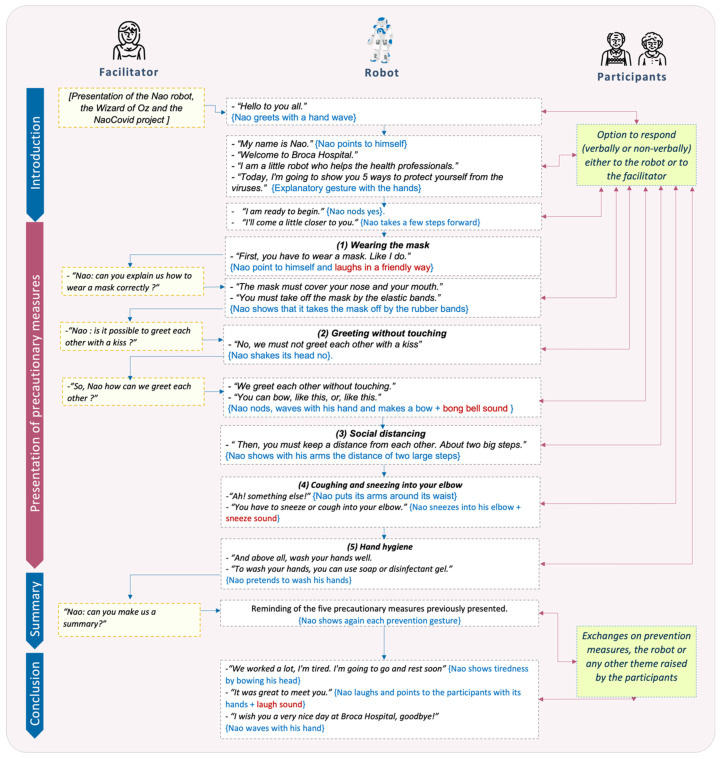
Outline of the interactive informational scenario for the presentation of COVID-19 precautionary measures.

The total length of the sequence was about 5 min. However, this duration could vary depending on the dynamic of the interaction with each participant. In terms of the scenario configuration, a waiting time was integrated between each sequence of the scenario to give the facilitator and the participants the possibility of interacting with the robot, if wished. A global presentation of the interactive information scenario is provided in Figure 3.

#### 2.2.3. Assessment Tools

The evaluation of this study was carried out using a questionnaire. To take into account the specificity of each group of participants included, two versions of the questionnaire were created: one for older patients and their accompanying persons, and the other for health professionals. The questionnaire included 13 items and was constructed in a simple way, providing clear instructions, and using Boolean questions (i.e., yes-or-no questions). For some of the questions, participants could argue their choices with free comments. In addition, participants who wished to do so could make suggestions for improving the robot-mediated intervention.

The first version of the questionnaire was intended for patients and accompanying persons. It was divided into the following two parts: the first part, distributed at the beginning of the activity, aimed at collecting the participants’ sociodemographic data (e.g., gender, age, profile, etc.), their self-rated knowledge of precautionary measures, and how they learned them. The second part, distributed at the end of the activity, aimed at evaluating their understanding and global perception of the activity, as well as its accessibility, usefulness, and acceptability. The different items of the questionnaire are listed in Table 1.

The second version of the questionnaire was intended for hospital professionals. It aimed at evaluating the interest of the activity for older adults and/or cognitively impaired people, for instance, whether the activity was understandable, accessible, and useful for the provision of health prevention information for these specific publics. The different items of this questionnaire are listed in Table 2.

We collected and examined all the free comments with the purpose of identifying key barriers and enablers to the feasibility, the acceptability, and the usefulness of the robot-mediated intervention. This analysis was also used for the suggestion of recommendations for the implementation of health promotion interventions using social robots as mediators.

### 2.3. Procedure

Prior to conducting each activity session, the activity facilitator checked that the tools (robot and computer to run the Choregraphe composition) were correctly loaded. For hygienic reasons, a disinfectant, a cloth, and a hydroalcoholic gel were also used to clean the robot. An Ethernet cable or a shared mobile connection was set up beforehand for the robot’s local network connection.

The objective of the activity and the evaluation procedures were orally presented to the different categories of participants recruited who agreed to participate in the activity. Afterwards, the participant(s) were invited to sit in the activity room to take part in the one-to-one activity session. Participants could be accompanied by one or two family caregivers. This individual or very small group setting was preferred so that all participants could fully enjoy the activity and interact easily with the Nao. Each participant was then given a written information note presenting the activity. If the persons confirmed their agreement to participate in the activity, a consent form to participate in the activity was given to them to be signed.

**Figure 4:**
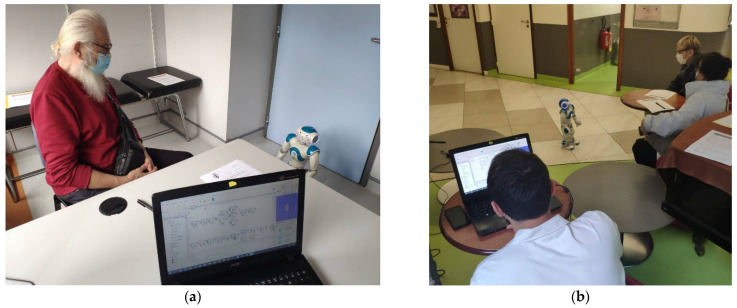
Robot-mediated activity for the promotion of precautionary measures: (**a**) Individual test session with a patient from the Memory Clinic of the Broca Hospital (**b**) Test session with a patient and a family caregiver at Broca Hospital.

Two researchers conducted the activity, a facilitator and a person who controlled the robot and the computer with the software. The facilitator’s role was to lead the session and provide help or additional information to participants when needed. The other person was in charge of managing the robot’s behaviour, using the Wizard of Oz technique [40], by initiating the different movement sequences of the Nao robot and responding to participants when they wanted to communicate with the robot (e.g., improvised verbal and non-verbal behaviour). The robot-mediated activity is shown on Figure 4.

Twenty participants in the geriatric rehabilitation day care hospital agreed to be filmed. From the video observations, we developed a short observation grid including the following verbal and non-verbal behaviours that could indicate participants’ engagement during the interaction with the robot: (1) Participant imitates the robot; (2) Participant laughs when the robot makes a joke; (3) Participant nods with his head to show his agreement to what the robot says; (4) Participant asks the robot to repeat the instruction; (5) Participant answers to the robot; (6) Participant maintains his gaze on the robot during all the activity; (7) Participant repositions or check that his mask is correctly positioned on their face, following the indications provided by the robot.

## 3. Results

### 3.1. Socio-Demographic Data of Participants

One hundred twenty height persons (n = 128) agreed to participate in the robot-mediated activity. However, the researchers stopped the intervention at its beginning for two older adults with major neurocognitive disorders who did not seem to understand the activity or participate in it in a satisfactory way. Finally, one hundred twenty-four persons (n = 124) participated in the entire activity and completed the questionnaire. In total, 68% (n = 84) of the participants were female and 32% (n = 40) were male.

The following three types of participants were included: older patients, accompanying persons, and hospital professionals. The composition of the groups in terms of size was rather equivalent, as 45 participants were older patients, 39 were accompanying persons, and 40 were hospital professionals. The average age of older patients was 81.6 years, 62.9 years for accompanying persons, and 44.6 years for hospital professionals. Of the total number of participants, 50% were under 65 years and 50% were over 65 years. The average age for all the sample was 63.7 years, with individuals ranging from 18 years to 93 years old. Socio-demographic data of the participants in the study are presented in Table 3.

None of the patients and accompanying persons had interacted with the robot before the present activity. Out of 33 professionals, 24% already knew the Nao robot before the present intervention.

### 3.2. Dimensions Addressed in the Evaluation

#### 3.2.1. Self-Rated Knowledge of Precautionary Measures

Results showed that 60% of the patients were familiar with the COVID-19 precautionary measures, 24% were vaguely familiar with them, and 16% of the patients were not familiar with them. Among the accompanying persons, 90% reported being familiar with these measures, 8% vaguely familiar, and 2% reported not being familiar with them. The different ways in which the precautionary measures were learned are reported in Table 4.

For patients and accompanying persons, the public media (e.g., television, radio, newspapers, and magazines) was the main source of information (55% for patients and 51% for accompanying persons). The family context and the hospital were also frequent sources of information. The results also showed that 15% of patients and 10% of accompanying persons answered “everywhere”, with these participants having the impression of having received the information on prevention and COVID-19 barrier gestures in different contexts without specifying a particular source of information.

#### 3.2.2. Acceptability of the Robot

As far as the acceptability of the robot was concerned, 98% of the patients and 97% of the accompanying persons stated that they enjoyed the activity with the robot: “*This robot is adorable with a beautiful head and a beautiful shape.*” (patient), “The *robot has a magical effect.*” (patient), “*I find Nao very nice, funny, extraordinary.*” (accompanying person). Respectively, 93% and 95% of the patients and accompanying persons felt confident during the activity. No participant left the activity because of fear of the robot, nor did they express a feeling of fear or discomfort with respect to the robot. The results also showed that 81% of the patients and 77% of the accompanying persons were willing to do an activity with Nao again*,* “*I would love to see it again, not necessarily to do an activity, but just to greet it.*” (patient).

#### 3.2.3. Overall Perception of the Activity: Duration and Content

93% of the patients and 97% of the accompanying persons stated that the duration of the activity was appropriate (e.g., many said that they did not see the time passing). Among hospital professionals, 20% thought that the activity could be too long for older adults, especially older adults with cognitive impairment.

Results also showed that 83% of the patients and 97% of the accompanying persons considered that they had understood the activity: “*The robot uses a simple vocabulary, it has a clear diction accompanying the gestures*” (patient), “*The activity is understandable by all.*” (accompanying person*)*. Among hospital professionals, 90% (n = 37) thought that the activity was understandable by older adults.

#### 3.2.4. Perceived Usefulness of the Activity

Regarding the perceived usefulness of the activity, 66% of the older patients and 46% of the accompanying persons answered that they had learned something about COVID-19 precautionary measures or about the interest in robot-mediated health promotion (e.g., “*It’s a good reminder of barrier gestures.*” (patient and accompanying person)*,* “*[I learned] that an activity with a robot can be very instructive.*” (accompanying person), “*I learned that with a robot, you can educate yourself.*” (patient), “*I learnt about social distancing.*” (patient), “*I learnt about taking off the mask by the rubber band.*” (patient), “*This activity complements the action of professionals.*” (accompanying person).

Out of 33 hospital professionals who answered the question, “Do you think the robot can be useful in transmitting preventive health information?” 97% (n = 32) answered positively. “*The robot has more impact on the patient than the usual professional advice.*” (professional), “*This activity should be done on a regular basis.*” (professional). A single professional who provided a negative answer insisted on the necessary presence of a human person for health prevention activities.

#### 3.2.5. Analysis of the Videos of the NAO Intervention

For the 20 participants who were filmed, the analysis of videos using the observation grid showed that 75% of them asked questions about the robot, 65% imitated the robot (e.g., saying hello back to the robot with their hand, pretending to sneeze in its own elbow), 25% asked the facilitator or the robot to repeat some instructions, 60% laughed (smiles were not counted because of the mask), 90% nodded during the intervention as if acknowledging the sense of the presentation, 85% responded to the robot (e.g., hello Nao, yes, absolutely, etc.), 80% kept their gaze fixed on the robot for the duration entire activity, and 25% repositioned or checked that their mask was correctly positioned on their face.

#### 3.2.6. Analysis of Key Barriers, Enablers, and Recommendations for the Implementation of the Intervention

Most participants made comments that entailed the identification of barriers and enablers for the robot-mediated intervention. They are reported in Table 5. Participants also suggested other potential robot-mediated activities such as the following:

- Making the robot available in a waiting room while waiting for test results to provide some distraction for patients and accompanying persons and reduce boredom;
- Offering various types of relaxation and leisure activities using the robot to help reduce anxiety and stress for patients and families who come to consult or visit;
- Organizing robotic mediated activities to deal with loneliness and isolation of patients in geriatric or paediatric departments;
- Using social robots to support healthcare workers and reduce their workload;
- Using social robots in the framework of rehabilitation therapies, (e.g., physical activity) or for monitoring vital functions;
- Integrating social robots into educational activities at school with younger audiences.

## 4. Discussion

This study had the following two objectives: (1) To develop an interactive informational activity to encourage the learning or recall of COVID-19 precautionary measures for older adults in geriatric facilities, using the humanoid robot Nao as a mediation tool, and (2) to assess the feasibility, acceptability, and perceived usefulness of the robot-mediated activity. The barriers and facilitators for this kind of intervention were also identified. In this section, we discuss the main results observed in this experimentation, describe its limits, and formulate some practical recommendations for the implementation of informational activities for health prevention using the mediation of a social robot.

### 4.1. The Feasibility of the Conception and Implementation of the Robot-Mediated Activity

Regarding the feasibility, in terms of the design process, we succeeded in developing and implementing an interactive informational scenario to present the main COVID-19 precautionary measures using the Nao robot. This experience confirms the flexibility of the Nao robotic platform to develop different sequences of behaviours associating the verbal and non-verbal dimensions.

However, some technological barriers were identified during the development of the activity. We found it difficult to implement certain gestures for the robot. For example, with Nao having only three fingers, it was not possible to make it wear its mask by itself (e.g., by manipulating the elastics that hold the mask behind the ears) or even to simulate the gesture (e.g., make the robot put its hands behind its head as if it was putting the mask on). Although the representation of such *iconic gestures* (i.e., visual representation of the object or action portrayed) by the robot was limited, we observed that it was possible to compensate to a certain extent for this limitation by trying to use *deictic gestures* (i.e., gestures used to point toward concrete objects or to call attention) to elicit the comprehension of the gesture within the framework of a discourse associated with it. In this case, we chose to program the robot to make a pointing gesture associated with the precautionary measure “wearing a mask correctly”, for which the robot showed the mask on its head using its hands. Our study confirms the findings from previous studies that reported the benefits of including different kinds of gestures (e.g., iconic, deictic, metaphoric, etc.) to support and enrich the robot’s speech in human–robot interaction (HRI) [41].

Another important issue regarding HRI was the understanding of the robot by the participants. One person did not find the content (i.e., the speech enunciated by the robot) of the activity easy to understand, and 25% of the participants asked the facilitator to repeat at least one instruction. This could be due to the robotic voice itself or to the sounds the robot makes when it moves, for instance when walking or moving its arms. These sounds may obscure the robot’s voice sometimes. These technical problems using robotic applications, particularly regarding speech recognition and production, have already been noticed in previous works [42,43,44]. For further experiences, tests could be carried out to identify which robot’s voice pitch, speed of dialogue, and sounds could be most suitable for an older adult audience and how best to adapt them to a Nao-type robot.

Another technical limitation was observed regarding the automation of the robot’s dialogues. When designing the activity, an autonomous interaction was planned between the participants and the robot. At the end of the session, the robot asked the participants if they had understood the prevention messages and if they had any other questions. Depending on the answer of the person (i.e., yes-or-no answer), the robot had to enunciate a predetermined sentence. During tests conducted in the laboratory, a rather quiet environment, the robot appeared to understand the user’s speech and respond accordingly. However, during a field test in the hospital waiting room, a noisier environment, this autonomous interaction was not possible since the robot could not understand users’ speech. Autonomous interactions were then removed and replaced by moments of improvised speech controlled by the person in charge of the robot (i.e., the Wizard of Oz).

The Wizard of Oz technique, used to control the robot in real-time, seemed effective for small-scale tests, but since it requires the participation of a professional, its use would be expensive for a permanent and/or wider implementation. A suggestion could be to create a digital application to launch the informational script with the complete staging, including the sequences of verbal and non-verbal behaviours, and an option for improvised simple dialogues and gestures. Such an application would allow the implication of a single facilitator instead of two, which could help with a wider implementation of robot-assisted activity sessions.

The feasibility of the intervention was also dependent on certain human factors. For instance, the presence of severe neurocognitive disorders in older adults was a limiting factor for their participation in the activity. The researchers stopped the intervention for two persons with major NCDs who did not seem to understand the activity or participate in it in a satisfactory way. This point has already been emphasized by other works [45,46], which have described that older adults with dementia may encounter significant challenges when interacting verbally with a robot, and, consequently, may show signs of confusion and/or ignore the robot. One option for the conception of health prevention information scenarios using robotic mediation for audiences with significant comprehension difficulties could be to use mainly the gestural dimension of the robot, thus limiting dialogues. Probably, designing a simpler activity that focuses only on a single instruction or measure could help to address this challenge as well.

Several factors favoured the feasibility of the intervention. The activity was carried out in good conditions. The environment was pleasant, calm, and favourable to the performance of the activity (e.g., provision of tables, space large enough to position a camera, ventilation). The activity was short and could easily be performed by older adults. As previously shown in the literature [25,47,48,49], we confirmed that the presence of a facilitator (animator) who co-animated the session with the robot was useful for several reasons: (1) by his encouraging and positive attitude, the facilitator invited the participants to interact with the robot; (2) the facilitator’s presence allowed participants to feel at ease with the robot; (3) the facilitator could give participants additional explanations about the activity or about the robot.

### 4.2. The Acceptability of the Robot-Mediated Intervention

Regarding the acceptability of the robot, the results from the questionnaires and the video analysis showed that most participants from the three groups (older patients, accompanying persons, and professionals) appreciated the intervention and showed strong engagement in the activity. Indeed, all the participants had at least one verbal or non-verbal interaction with the robot. Some of them responded directly to the robot by saying hello or greeting with their hands. Others imitated the robot when the latter showed that it was necessary to sneeze into its elbow or even put its mask over its nose and mouth. These verbal and non-verbal responses observed contribute to validating the hypothesis that the use of the robot promotes engagement during health prevention activities. Our results confirm the results of other works as well, which found that the Nao robot captured older adults’ attention and facilitated communication [28,30,50]. As in other studies [51], the pleasant aspect of the robot played a key role in its acceptability in our experimentation. The robot’s social presence, friendly personality, and harmless appearance were acknowledged by participants in our study as factors that contributed to the acceptance of the activity. These indicators are crucial for the acceptability of robots, as already underlined in the Almere model, a model of technology acceptance specifically developed to test the acceptance of assistive social agents by elderly users [23,52,53]. It is also likely that the joint involvement of the accompanying person in the robotic intervention certainly played an important role in reassuring the participants and motivating them to engage in the activity.

### 4.3. Usefulness of the Intervention

Before taking part in the robot-mediated activity, most of the participants in the three groups (patients, accompanying persons, and professionals) declared having learned the COVID-19 precautionary measures by different means. Nevertheless, some persons, particularly older patients (40%), declared a lack of information regarding COVID-19 prevention. Several authors have noted that common disorders observed in older adults (e.g., sensory deficits, depression, and cognitive impairment) may negatively impact access to and use of health information [54,55]. Since some of the older patients who took part in our activity probably had cognitive disorders (e.g., Alzheimer’s disease or related disorders), their condition may have made it more challenging for them to understand COVID-19 disease and to learn precautionary measures before attending our activity. This observation confirms previous data regarding the specific challenges faced by older adults with NCDs in understanding and following recommendations from public health authorities to reduce COVID-19 disease transmission [6,8,56].

For older adults with NCDs, one of the issues that make access to health information more difficult is the use of complicated language and/or many medical or technical terms [57]. D’Cruz and Berrjee (2020) [6] have recommended that health information delivered to persons living with dementia must be performed slowly, in short and simple sentences, using frequent pauses, and ideally using audio-visual aids. In accordance with these recommendations, for the informational scenario using the social robot, we developed short and simple messages to present COVID-19 precautionary measures that were announced by the robot, and we added a corresponding gesture to facilitate understanding. The results we obtained in the questionnaire and in the video analysis showed that this association (speech and gesture) was useful for enabling the understanding of the prevention message. Robot-mediation appeared to play a key role in facilitating the comprehension of the information, particularly 83% of older patients declared having understood the activity. Professionals and accompanying persons also felt that the activity was easy to understand and, to this extent, would be appropriate for older people with cognitive impairment.

We formulated the hypothesis that the activity with the Nao favoured the engagement of the participants in a health prevention activity. Our results showed that participants found that the robot was an enjoyable and engaging tool. Most participants accepted the robot as a mediating tool for the transmission of health information. This kind of support may be useful as well for other audiences with intellectual or developmental disorders who may struggle understanding traditional health information supports [57,58].

The results of our study showed that humanoid robots, such as Nao, can be used as interactive, engaging, and useful supports to promote health information for older adults. Other technologies have been used to improve health literacy in this population. For instance, some works [59,60,61] have shown that embodied conversational agents were practical supports to present healthcare information (e.g., providing health tips, nutrition coaching) to older adults. Within the context of the COVID-19 crisis, some works have described the use of social robots for detecting the absence of a facial mask or improper facial mask-wearing and providing corresponding warnings [62,63]. However, to our knowledge, the present work is the first study using a social robot to deliver accessible health information related to COVID-19 in geriatric care settings.

### 4.4. Ethical Issues

Ethical questions are important and need to be addressed when implementing a robot-mediated intervention in the field of healthcare. A literature review of publications on the ethics of the use of robots for geriatric care [64] described, among others, a *deontological* approach for ethical analysis that involves the following three main points: (1) respecting the autonomy and dignity of older adults when implementing robot-assisted care; (2) identifying the risk of deception when introducing care robots that may pretend to be something they are not (e.g., being companions, being real social actors), making the best to communicate about them in a truthful way; (3) considering the risk of social isolation that the use of social robots may entail for older adults, promoting instead care practices that favour social connectedness.

In our study, we noted a very limited number of comments by participants linked to ethical issues. Some reasons why these issues were not further discussed or pointed out by the participants may be the following:

- The fact that everyone was free to participate or not in the activity;
- Each person who accepted to participate in the activity, could decide to interact with the robot at the level of involvement he or she wanted (e.g., only observing, responding verbally or non-verbally to the robot, initiating an interaction with the robot, etc.);
- The nature of the robotic tool and the presence of the Wizard of Oz person who handled the robot were clearly explained;
- A methodological limitation in that, overall, ethical issues were not explicitly addressed in the evaluation protocol.

Nevertheless, one older patient noted that he did not want to trust the robot, a remark that can refer to the first deontological point regarding the respect for the autonomy of the person (i.e., not wishing to use a robot for any reason), but also to the second point, about not considering the robot as a reliable entity. In another case, an accompanying person underlined that the robot should not replace the human presence, illustrating the third deontological point. These views and opinions were heard and respected.

Participation in a robot-mediated activity should always be voluntary and enjoyable for the participant. In this study, the free nature of the participants encouraged the respect for the autonomy of the person about his choice of taking part in the activity or not. Moreover, it is worth keeping in mind that the robot-mediated activity was conceived in such a way that the facilitator was highly involved in the scenario. This choice was made to reduce the concerns about the replacement of healthcare workers by robotic interventions. We consider that associating the professional with the implementation of activities using social robots is a key factor for the smooth and respectful integration of this form of mediation into professionals’ care practices. With this aim, the sequences of dialogue and actions in our script were structured to alternate the intervention of the facilitator and the robot. With respect to this point, our study is in line with the “care triangle” approach for robot-mediated care interventions, which we have implemented in a previous work [25] and that has been widely described by Parviainen and Pirhonen (2017) [65]. In this kind of approach, the different roles of the caregiver, the care receiver, and the robot are defined in advance to complement each other in robot-mediated interventions.

### 4.5. Recommendations for Robot-Mediated Intervention for Health Prevention

The results of this study enabled us to suggest some recommendations for the development and implementation of robot-mediated activities to improve the accessibility of health information for older adults as follows:

Preparation of robot-mediated activity sessions

- Define the target population as follows: Inclusion in this kind of activity must privilege older adults who are cognitively healthy or have mild or moderate cognitive impairment, according to the type of information to be provided and the objectives of the activity. The participation of persons with significant communication and/or comprehension impairments should be examined on a case-by-case basis;
- Identify audience needs as follows: Examine the needs of older adults in terms of health information (for instance, precautionary measures for COVID-19 infection) and prepare the contents of the message accordingly;
- Develop HRI scenarios as follows: Develop scenarios combining robot’s speech and gestures. Prepare short, precise, and simple key messages and associate them, if possible, with embodied cues (i.e., a set of gestures and other nonverbal behaviours that convey information and support the speech). Sound effects can be useful to encourage participants’ attention and motivation;
- Preparation of the robot as follows: Program the robot in advance and adapt its voice, speech, and volume for people with potential hearing loss. Make the objects that support the informational message visible and identifiable (for instance, the mask in the case of the COVID-19 precautionary measure scenario). Plan to start the activity with the robot located at 2.50 m away from the participant to allow for a gradual familiarisation with the robot’s presence. After introducing itself, the robot can approach the participant to facilitate visual access to the gestural explanations, up to 1.50 m. Before each activity session, verify the technical aspects that are necessary for the correct use of the robot (e.g., availability of up-to-date software, level of charge batteries, wifi connection, hygiene procedures, etc.);
- Feasibility/acceptability/usefulness testing as follows: Test the feasibility, acceptability, and usefulness of the scenarios with some older adults and implement necessary adaptations to the activity before proposing a large-scale deployment of the activity.

Conduct and assessment of robot-mediated activity sessions

- Create a positive context as follows: Create a pleasant atmosphere between the participant, the facilitator, the Wizard of Oz (if available), and the robot.
- Check consent as follows: Before starting the activity, explain the context and objectives of the session, make sure that participants feel at ease with the robot, and verify that they agree to take part in the activity at its beginning and throughout its course;
- Facilitate understanding and learning as follows: The facilitator plays a key role in promoting participation in the activity session. To favour participants’ understanding and learning, the facilitator should articulate well and observe a sufficiently slow rhythm, check participants’ reactions to verify understanding throughout the session, and rephrase or imitate what the robot says or does when needed. A few simple questions addressed to participants can be integrated throughout the presentation to keep their attention and to encourage the retention of the message;
- Assess pre-and post-learning as follows: in cognitively fit older adults who appreciate challenges, assess knowledge in the health domain before and after the robot activity.

### 4.6. Limitations of the Study and Future Work

The study has some limitations that should be considered when interpreting the results. First, although some participants in our study (the older patients’ group) obviously presented cognitive and hearing disorders, the assessment of these health conditions was not performed in our protocol. It is noteworthy that cognitive, hearing, or visual impairments can affect the interaction of users with the robot, influence the activity impact, and, therefore, should be considered in future studies.

Second, we did not ask the patients and accompanying persons about their prior experiences regarding social robots. Some participants mentioned that they had already seen the Nao robot on television or in other contexts, but most participants did not comment on this point. Therefore, one cannot exclude that the results showing good acceptance of the Nao robot were biased by novelty effects. In future studies, it would be useful to include questions about participants’ prior knowledge of social robots to assess the potential novelty effect of the Nao robot.

A third limitation of our study is that a formal evaluation of health literacy in the domain of precautionary measures in COVID-19 infection before and after the robotic intervention was not conducted. In our study, most older patients declared having understood and learned the prevention-related information during the activity, but one could argue that this positive answer carries a bias of social desirability [66]. Future studies would benefit from an assessment of participants’ health literacy, for instance, using the Test of Functional Health Literacy in Adults (TOFHLA) [67]. Another suggestion to improve the quality of the evaluation of this intervention could be to investigate the usefulness of the training by comparing knowledge and skills to accomplish barrier gestures before and after the robotic intervention in older adults, with an assessment made by both the participants themselves and health professionals. In further work, we could also study the understanding and benefits of the robot-mediated activity for the transmission of health information according to different participants’ profiles (e.g., age, pathologies, cognitive functioning, health literacy, etc.), thus making it possible to accurately evaluate the usefulness of the robotic activity for different audiences. It might also be interesting to compare the learning effects when the health information is provided by a health professional, directly or using an educational video, and when the Nao robot is used for this purpose, to assess which would be more effective.

A fourth limitation concerns the choice of the robot used in this study. The Nao robot is small and, in some ways, fragile. This can present some challenges if used in crowded environments or with a lot of people moving around (i.e., the robot could be unseen and accidentally hit or kicked). In our study, the fact that participants were seated during the demonstration, and that the experience was conducted in a one-to-one or very small group format, ensured its execution without any incidents of this type. However, the implementation of the activity in more complex environments needs to be examined in terms of safety for both the participants and the robot.

Finally, in future studies on health information delivery in older adults, we could incorporate artificial intelligence. For instance, the robot could be programmed to verify if the health prevention information has been correctly learned (e.g., using robot sensors to check that hand sanitiser has been correctly applied or to check that the mask has been correctly worn). It would also be useful to develop robot-mediated interventions that convey health information to a wider spectrum of people and regarding other health education topics.

## 5. Conclusions

Understanding and compliance with preventive measures have been a major issue during the COVID-19 pandemic. The results of this study showed that a social humanoid robot can be used as an innovative communication tool to promote COVID-19 precautionary measures in older adults with and without cognitive impairment. The importance of working to improve health literacy among older adults and individuals with cognitive impairment, intellectual disability, or mental illness is, nowadays, widely acknowledged. This new intervention modality for health prevention can be added to the existing range of accessible support for audiences with special needs.

## Figures and Tables

**Table 1:** Dimensions and related issues addressed in the patient and accompanying persons questionnaire.

Dimensions	Questions *(Answer Modality)*
Socio-demographic data	Gender *(Check box: Female; Male)*
	Year of birth *(__|__|__)*
	Profile *(Check box: patients; accompanying persons; professional category for health professionals)*
Knowledge of precautionary measures	Do you know the different precautionary measures to protect yourself from COVID-19 infection? *(Yes/Moderately/No)*
	Where did you hear about precautionary measures? *(Free comments)*
Perception of the activity	Did you find the robot-mediated activity long? *(Yes/No)*
	Did you find the content of the activity easy to understand *(Yes/No, why?/free comments)*
Perceived usefulness of the activity	Did you learn anything about precautionary measures *(Yes/No)*
	Did you learn anything about technology? *(Yes/No)*
	Did you learn anything else? *(If so, what did you learn?/free comments)*
Acceptability of the Nao robot	I enjoyed the activity *(Yes/No, why?/free comments)*
	I felt confident in this activity *(Yes/No, why?/free comments)*
	I would like to see the Nao robot in another activity *(Yes/No)*
Other	Do you have any other comments or information to share with us? *(Free comments)*

**Table 2:** Dimensions and related issues addressed in the questionnaire for hospital professionals.

Dimensions	Questions *(Answer Modality)*
Socio-demographic data	Gender *(Check box: Female; Male)*
	Year of birth *(__|__|__)*
	Profession *(Free comment)*
Knowledge of the Nao robot	Did you already know the Nao robot? *(Y/N, If yes, in what context?)*
Acceptability of the NAO robot	Did you enjoy the activity with the NAO robot? *(Y/N/free comments)*
Perception of the activity	Do you think that the activity is too long for older adults and/or persons with cognitive impairment? *(Y/N/free comments)*
	Do you think the activity is understandable for older adults and/or persons with cognitive impairment? *(Y/N/free comments)*
Perceived usefulness of the activity	Did you learn anything new about precautionary measures? *(Y/N/free comments)*
	Did you learn anything about technology? *(Y/N/free comments)*
	Did you learn anything else? *(If so, what did you learn?)* Do you think that the robot-mediated activity can be useful for the transmission of preventive health information? (*Y/N, why?/free comments)*
Others	Concerning the overall activity with the Nao robot, do you have any suggestions for improvement? *(Free comments)*
	Do you have any other comments or information to share with us? *(Free comments)*

**Table 3:** Socio-demographic data of participants.

Variables	Modalities	Patients	Accompanying Persons n (%)	Health Professionals n (%)
Count	-	45 (36%)	39 (32%)	40 (32%)
Gender	Male	15 (35%)	14 (35%)	11 (28%)
	Female	30 (65%)	25 (65%)	29 (72%)
Age	64 y/o or younger	1 (02%)	21 (54%)	40 (100%)
	65–80 y/o	13 (29%)	11 (28%)	0
	80–95 y/o	31 (69%)	7 (18%)	0
	Average	81.57	62.90	44.56

**Table 4:** Participants’ (patients and accompanying persons) results on self-rated knowledge of COVID-19 precautionary measures.

Variables	Modalities	Patients n (%)	Accompanying Persons n (%)
Do you know the different precautionary measures?	Yes	27 (60%)	35 (90%)
	Partly	11 (24%)	3 (08%)
	No	7 (16%)	1 (02%)
Where did you hear about them?	Public media	25 (55%)	20 (51%)
	Family context	6 (13%)	6 (15%)
	Work	0 (0%)	6 (15%)
	Hospital	7 (15%)	3 (8%)
	Everywhere	7 (15%)	4 (10%)

**Table 5:** Barriers and enablers for robot-mediated intervention.

Barriers	Enablers
**Feasibility**	
**Robot’s connection time** “*The robot is a bit slow to connect.*” (professional) **Robot’s voice, flow** “*The robot’s voice is not very understandable.*” (patient) “*The artificial voice of the robot can be annoying for older adults.*” (professional) **Robot ‘s annoying noises** “*The robot makes some annoying engine noises.*” (professional) “*The footsteps of the robot are unpleasant to hear.*” (patient) **Robot’s limited gestures** “*The robot shows Interesting but limited gestures.*” (patient) **Robot’s small size** “*The size of the robot a bit small if you want to communicate with it standing up.*” (professional) **Accessibility issues** “*People with hearing impairment may have difficulties to understand the robot.*” (patient) “*The activity may be difficult to follow for people with attention difficulties or other neurocognitive disorders*” (professional) “*The activity is satisfactory for patients with mild cognitive impairment but more difficult for patients with moderate or severe cognitive deficits.*” (professional)	**Presence of a facilitator** “*It is easier to understand the activity with a little additional explanation from the animator.*” (patient) “*I enjoy the friendly atmosphere created by the animator who conducted the activity*” (accompanying person)
**Acceptability**	
**The robot may generate anxiety** “*The robot is not very reassuring.*” (accompanying person) “*I don’t want to trust the robot.*” (patient) **Fears of technology replacement** “*The robot does not replace the human person.*” (accompanying person)	**The robot does not generate anxiety** “*I feel good and at ease with this robot.*” (patient) **Robot engagement** “*The robot captivates me; it is motivating and exciting.* ”(patient) “*The patients are engaged in the robot activity*” (professional) **The novelty of the activity** “*I discover the capabilities of a robot, this a novelty for me.* ”(patient) “*This new activity changes from the usual explanations* ”(patient) “*An original way of explaining things*.”(accompanying person) **Robot social presence** “*It is adorable this robot, it has a beautiful head, beautiful shape, and he makes you laugh.*”(patient) “*Nao has an expressive gaze and seems very friendly.* ”(patient) “*Nao t looks like a friendly child.*” (accompanying person) **Robot sociability perception** “*Nao seems very benevolent and sympathetic.* ” (patient) “*We want to talk and communicate with it.*” (patient) “*You feel like talking to him.*” (accompanying person)
**Usefulness**	
**Lack of information in the scenario** “*The scenario lacks images presenting the hygiene products.*” (accompanying person) “*The activity lacks information about the need to ventilate rooms, for instance opening windows*”. (professional) “*The need to keep a distance between people is not well explained.*”(patient)	**Well-structured and simple activity facilitating learning** “*The messages are short, precise.*” (patient) “*The message is simple and direct.*” (professional) “*The gestures are very useful.*” (patient) “*This activity is motivating and entertaining.*” (accompanying person) **The activity stimulates cognitive functions** “*The activity attracts attention, stimulates sensory and executive functions.*” (professional) **The activity can be adapted to various contexts** “*This activity is suitable for various audiences and in different contexts (hospital, school).*” (patient)

## Data Availability

The data presented in this study are available on request from the corresponding author.
